# Acupuncture effect on digestion in critically ill postoperative oral and hypopharyngeal cancer patients: A protocol for double-blind randomized control trial

**DOI:** 10.1097/MD.0000000000016944

**Published:** 2019-08-30

**Authors:** Eyal Ben-Arie, Pei-Yu Kao, Wen-Chao Ho, Yu Chen Lee

**Affiliations:** a Graduate Institute of Acupuncture Science (Collage of Chinese Medicine) China Medical University; b Division of Thoracic Surgery, Department of Surgery; c Surgical Intensive Care Unit; d Department of Public Health, China Medical University; e Department of Acupuncture, China Medical University Hospital; f Chinese Medicine Research Center, China Medical University; g Department of Nursing and Graduate Institute of Nursing, Asia University, Taichung, Taiwan.

**Keywords:** acupuncture, critically ill, digestion, hypopharyngeal cancer, intensive care, intensive care unit, oral cancer

## Abstract

**Introduction:**

Head and neck cancer patients are at a high risk to suffer from malnourishment, a risk that increases in postoperative condition and with the use of enteral nutrition (EN). Until now patients who are suffering from indigestion in the intensive care unit (ICU) received treatment in the form of prokinetic drugs, drugs that can lead to serious side effects and only can partially improve digestion functions. Acupuncture was used successfully in several clinical trials to improve postoperative indigestion in cancer patients without any reported adverse events. The study aims are to investigate acupuncture effect in combination with prokinetic drugs in the treatment of indigestion in postoperative oral and hypopharyngeal cancer patients in the ICU.

**Methods:**

Single-center, double-blind randomized control trial will compare between 2 equal groups. A total of 28 patients that will meet the inclusion criteria: age 30 to 80, postplastic surgery for oral cancer or hypopharyngeal cancer, developed feeding intolerance 2 times in the first postoperative day, Apache score <20, and needed EN. Patients will be randomly divided (1:1) into treatment group or control group for 3 treatments in 3 days along with routine ICU treatment. The main outcome measurement will be the number of days a patient needs to reach his total energy expenditure.

**Expected outcome:**

The results will shed light on the effectiveness and safety of acupuncture in a double-blind design treating postoperative ICU cancer patients. In addition, the study presents a revolutionary double-blind design that if, will prove as successful might influence the way double-blind acupuncture studies are performed today.

**Other information:**

The study will be conducted in the surgical ICU department, of China medical university hospital, Taichung 404, Taiwan. The study is conducted on stable ICU patients and is anticipated to have minimum risk for adverse events. Patients enrollment and data collection will start from May 15, 2019. The study expected completion time: June 2021.

## 1 Introduction

Up to 80% of head and neck cancer patients will suffer malnourishment during the course of the disease, malnutrition can increase the risk of postoperative complications as well as the rate of mortality.^[1]^ Postoperative oral and hypopharyngeal cancer patients will usually lose the ability of oral feeding as a result of the surgery, a fact that when combined with chemotherapy and radiotherapy will only increase malnutrition risk.^[2]^ Postoperative intensive care unit (ICU) patient often required parenteral nutrition or enteral nutrition (EN) to prevent malnourishment. EN is considered as the superior nutrition choice that assists the maintenance of gut mucosal integrity, improved utilization of nutrients, reduced respiratory infections, and hospital total stay.^[3,4]^ Unfortunately EN carry a number of side effects such as delayed gastric emptying, diarrhea, constipation, reflux, nausea, vomiting, gastrointestinal (GI) bleeding, contamination, and EN feeding tube clogging.^[5–9]^ To prevent the side effect of EN, prokinetic drugs are often used. A recent systemic review and meta-analysis found that although prokinetic drugs can reduce high gastric residual volume, prokinetic drugs failed to decrease vomiting, diarrhea, mortality risk, and length of stay in the ICU.^[10]^ Metoclopramide, a popular and commonly used prokinetic drug, can lead to serious neurological and cardiac adverse reactions.^[11]^

Acupuncture is an ancient treatment that was used for thousands of years and is considered to be a safe treatment when applied by a certified acupuncturist.^[12–14]^ Acupuncture is rarely a part of the complementary treatment that is used in the ICU. A few studies used acupuncture in the ICU without any adverse events.^[15–18]^ A review investigating acupuncture for postoperative delayed gastric emptying patients including abdominal surgery, esophageal cancer, and laparotomy found promising effectiveness of acupuncture when comparing to prokinetic drugs.^[19]^ Two additional studies suggest that electrical acupoint stimulation might be superior to prokinetic drug usage in the treatment of delayed gastric emptying in EN patients in the ICU resultant from stroke or traumatic brain injury.^[7,20]^ Another study proves that acupuncture, along with Chinese herbal medicine, can decrease acute GI injury incidence in elderly patients with severe sepsis.^[21]^

As a control treatment, we will use nonspecific acupuncture, which uses “real” acupuncture but the points selected have no relation to improving digestion function. We believe that the use of nonspecific acupuncture might enable proper blinding to the acupuncturist. As far as we know, there is no double-blind randomized controlled study that investigates the effect of acupuncture on total energy expenditure (TEE) days and other accompanying digestive signs present in postoperative oral and hypopharyngeal cancer patients receiving EN. The goal of our study is to conduct a double-blind randomized controlled trial to investigate the effect of acupuncture on treating indigestion factors of postoperative oral and hypopharyngeal cancer patients receiving EN in the surgical ICU; furthermore, we hypothesize that specific acupuncture (treatment group) will have a greater beneficial effect on digestion indicators when compared with the control.

## 2 Methods

### 2.1 Design and setting

**Figure 1:**
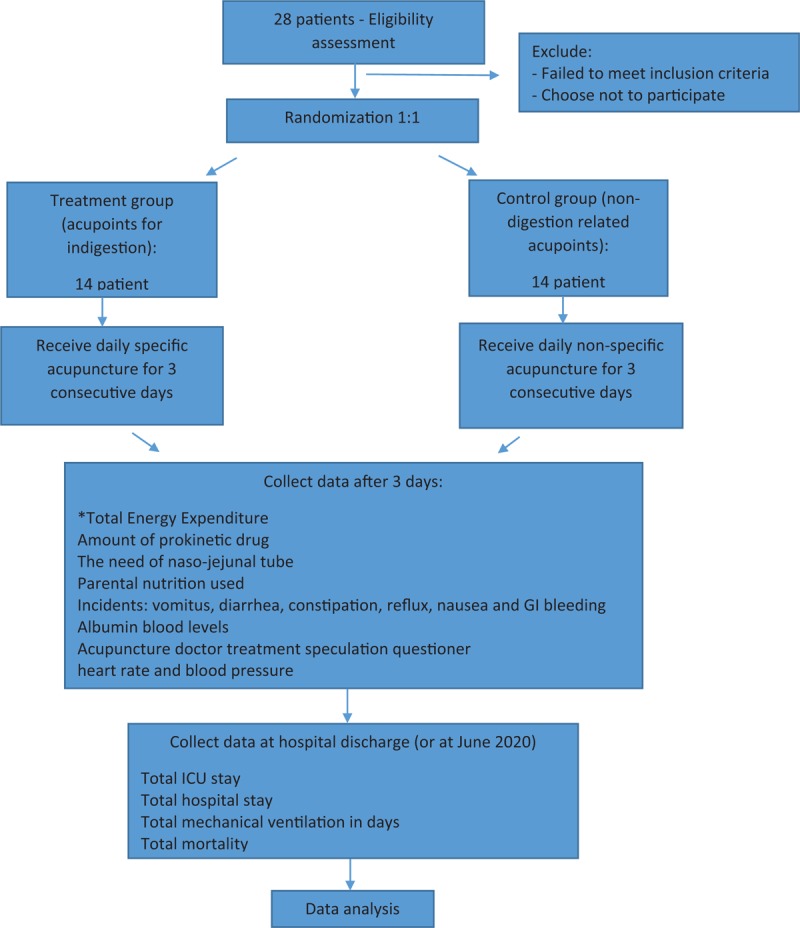
Study flow chart. A computer-generated randomization list prepared by study nurse will randomize 28 participants to either receive acupuncture with digestion-related acupoints (treatment group) or to receive acupuncture with nondigestion-related acupoints (control group). Each group will include 14 patients. Acupuncture intervention in both groups will take place on for 3 consecutive days for a total of 3 treatments. Patients follow-up will be completed on June 2021. ^∗^ Main outcome measurement.

Our study is a single-center, parallel arm, double-blind randomized control trial that will take place from May 2019 until June 2021. Our study will be performed in the surgical intensive care department of China Medical University Hospital in Taichung city, Taiwan. Our trial was approved by the Ethics Committee of China Medical University Hospital (CMUH108-REC2-037) and registered study protocol on www.clinicaltrial.gov (NCT03934294). The goal of our study is to assess the efficacy of acupuncture in treating indigestion and accompanying indigestion symptoms of surgical ICU patients. This will be achieved through analysis of 2 parallel groups; treatment group (ACU): specific acupuncture treatment alongside routine ICU treatment; control group (CON): nonspecific acupuncture treatment with routine ICU treatment (Fig. 1).

An informed consent form will be filed and signed by the legal representative of the patient before the initiation of our study. Patients will be randomly divided into 2 equal groups to ACU, and CON. Two qualified acupuncture doctors with at least 2 years of experience, from the acupuncture department of China medical university hospital, will perform the ACU and the CON treatments, ICU routine treatment will be carried out by ICU staff in all groups (ACU, CON). Prokinetic drug treatment will be prescribed by an ICU medical doctor if needed in all study groups. Researchers, statisticians, doctors, acupuncturists, and patients along with nurses (with the exception of study nurse) will be blinded to the patient allocation. Patients’ assessment will be done on a daily basis by ICU medical doctors and ICU nurses who will be blind to the patients’ allocation.

### 2.2 Participants

A total of 28 patients that required EN and admitted to the surgical intensive care department in china medical hospital for estimated ICU duration stay of at least 3 days will be enrolled in this study if the patient met the study inclusion criteria and after patient's legal representative will sign an informed consent form.

### 2.3 Inclusion criteria

- Age 30–80
- Apache score <20
- Patients needed EN
- Patients who developed feeding intolerance 2 times in the first postoperative day.
- Postplastic surgery, including oral cancer or hypopharyngeal cancer

### 2.4 Exclusion criteria

- Coagulopathy
- Prolong prothrombin time activated partial thromboplastin time >4 times
- Thrombocytopenia—low platelet count
- Clinically unstable: receiving 2 inotropic agents or Fraction of Inspired Oxygen >70%
- Estimated ICU stay <3 days

### 2.5 Recruitment strategies

Patients recruiting will be done from May 2019 to July of 2021 or until a total of 28 patients will be recruited. A study personal (KP-Y) that is responsible for communicating with patient's families and legal guardians will inform the patients family of the study along with its potential pros and cons and will ask the patients legal guardian to fill an informed consent form in case of willingness to join the study.

### 2.6 Informed consent

The study characteristics, objectives, risks, and benefits along with details on patient's rights according to the declaration of Helsinki will be explained to the patient's legal guardian by a study personal (KP-Y). In the event of willingness to enroll in the study, an informed consent form will be signed by the patient's legal guardian that can decide on withdrawal from the study at any moment. In case of withdrawal from the study, the patient's available data will be reserved for the final analyses (for the full informed consent form see Supplementary Information).

### 2.7 Randomization and allocation concealment

**Figure 2:**
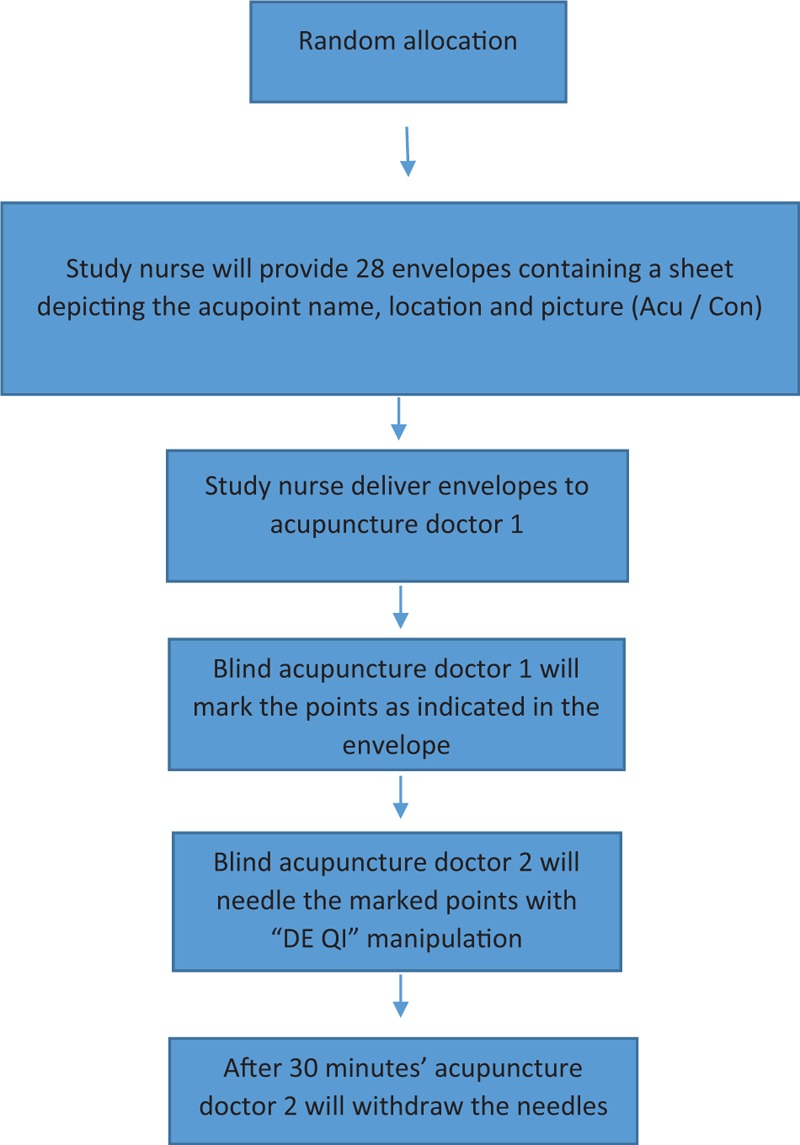
Blinding flow chart. Patients and acupuncturist blinding process: after random allocation study nurse will provide 14 nontransparent sealed envelopes containing acupoint name and picture for both treatment and control groups for a total of 28 envelopes. Acupuncture doctor 1 will receive the envelope, will mark the acupoints on the patient skin with a green sticker and will leave the room. Acupuncture doctor 2 will enter the room and needle the marked acupoints with “DE QI” manipulation. After 30 minutes’ acupuncture doctor 2 will withdraw the needles.

Patients will be randomized to 1 of 2 groups by study nurse (EB-A): ACU/CON using a computer-based simple random sampling with 1:1 ratio without stratification using the IBM SPSS statistics version 22 software (SPSS Inc, Chicago, IL). A number (1–28) will be affiliated to each patient at the time of ICU admission and after achieving informed consent. The number and patient name will be written down on a nontransparent envelope by the study nurse. The study nurse will provide an envelope containing a sheet depicting the acupoint name, location, and picture (ACU/CON) to the acupuncture doctor 1. Acupuncture doctor 1 (blind) will mark the acupoints on the patients’ body with a green sticker and leave the room, and then acupuncture doctor 2 (blind) will enter the room and perform acupuncture on the marked points. Thirty minutes after needle insertion, acupuncture doctor 2 will withdraw the needles. Acupuncturists, ICU nurses, ICU doctors, researchers (except EB-A) and statisticians will all be blind to group allocation and to the meaning of the patient's numbers until all acupuncture treatments have been completed. The 2 acupuncturists will also be blind to the treatment goals. Both acupuncture doctors will not be allowed to speak with the patients or themselves about the study goals or patients’ allocation (Fig. 2).

### 2.8 Interventions

Patients of the surgical intensive care department in China medical university hospital Taiwan, which have met the inclusion criteria, will be randomized and divided into 2 groups and receive specific acupuncture treatment with routine ICU treatment (ACU); nonspecific acupuncture with routine ICU treatment (CON). The patients will receive everyday treatment for 3 days for a total of 3 treatments. The treatment will start after 2 failed tube feeding sessions in both groups.

### 2.9 Treatment group (ACU)

**Figure 3:**
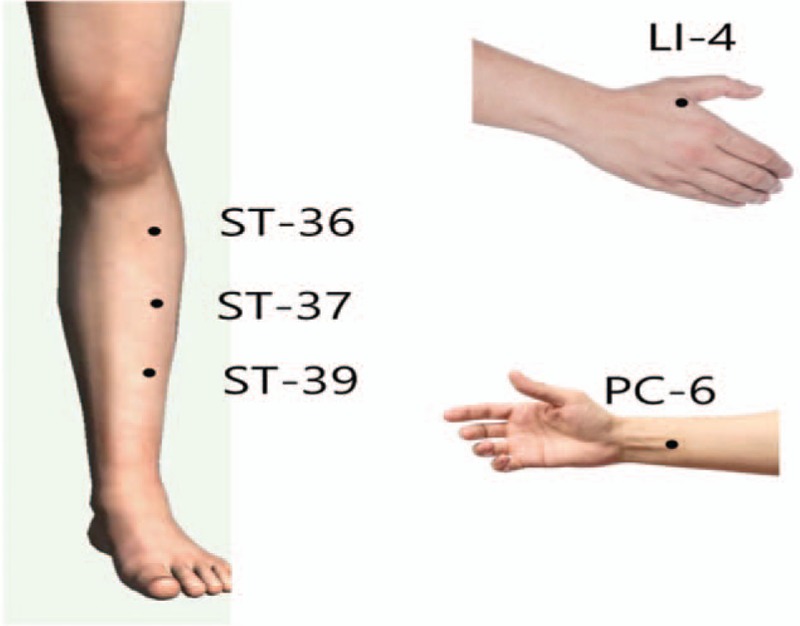
Acupoints for indigestion as selected in the Treatment group (ACU). The following acupoints will be applied for the treatment group (ACU). The acupoints indications in this group are specific to treat indigestion related conditions. ST36 (Zusanli), ST37 (Shangjuxu), ST39 (Xiajuxu), PC6 (Neiguan), and LI4 (Hegu).

In addition to routine ICU treatments, patients in the specific acupuncture group (ACU) will also receive daily bilateral traditional Chinese medicine style acupuncture on the following acupuncture points: ST36 (Zusanli), ST37 (Shangjuxu), ST39 (Xiajuxu), PC6 (Neiguan), and LI4 (Hegu). The acupoints indications in this group are specific to treat indigestion-related conditions (Fig. 3). The treatment will take place once a day, over 3 days, for a total of 3 treatments. A total of 10 needles will be used in each session. Acupuncture treatment will be performed with sterile needles manufactured by “Yu Kuang” acupuncture needles 40 mm with 30 G.

After receiving an envelope containing the selected points from the study nurse, acupuncture doctor 1 (blind) will mark the points on the patients’ body with a green sticker and leave the room. Acupuncture doctor 2 (blind) will disinfect the marked acupoints with an alcohol pad containing 70% alcohol and will perform acupuncture on the marked points with perpendicular needle insertion into the muscle layer, followed by up and down manipulation and 180° rotation of the needles in both directions to generate a “De Qi” sensation. During the session the patients will stay in a supine position. Needle retention time will be 30 minutes. The needles will be withdrawn by acupuncture doctor 2, who will not be allowed to communicate with the patients. At the end of the treatment, acupuncture doctors 1 and 2 will be asked to guess if they treated the specific or nonspecific acupuncture group to assess and maintain the integrity of the blindness in the study.

### 2.10 Control group

**Figure 4:**
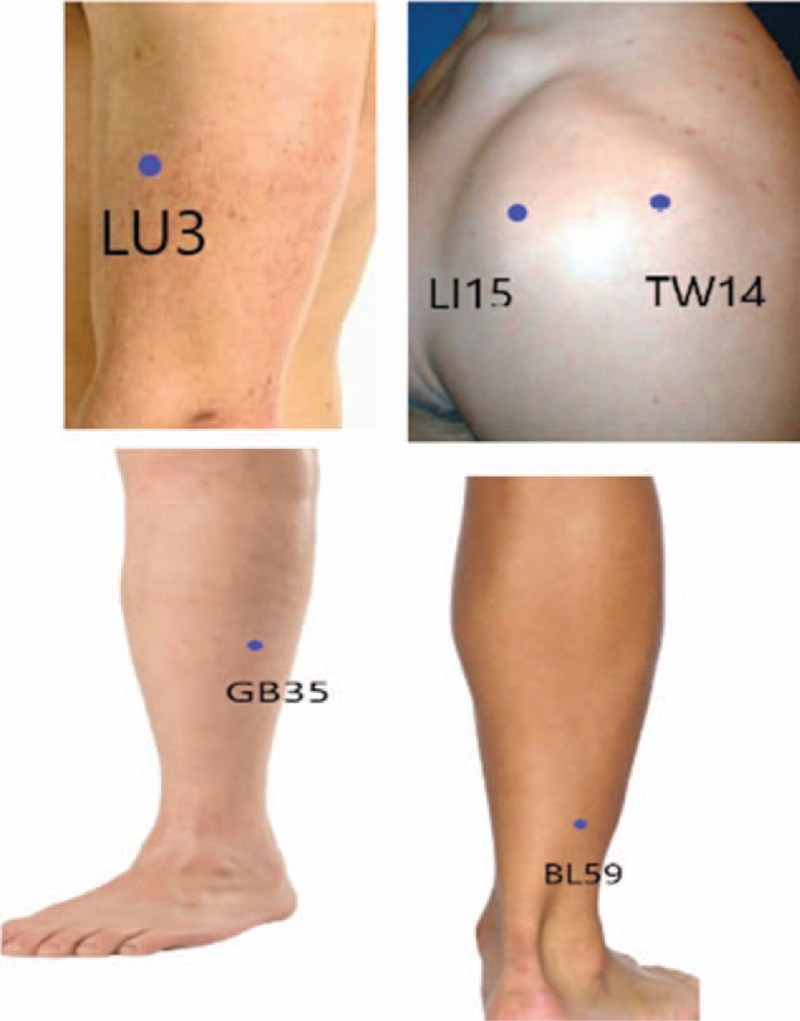
Acupoints as selected in the control group (CON). The following acupoints will be applied for the CON. The selected control points are not indicated for the treatment of digestion-related pathologies and are not reported to improve digestive function. LI 15 (Jianyu), SJ 14 (JianLiao) LU3 (Tianfu), GB35 (Yangjiao), and BL 59 (Fuyang).

Patients’ in the nonspecific acupuncture group (CON) will receive routine ICU treatment and a total of 3 daily indigestion-related traditional Chinese medicine style acupuncture treatments at the following acupoints: LI 15 (Jianyu), SJ 14 (JianLiao), LU3 (Tianfu), GB35 (Yangjiao), BL 59 (Fuyang). The selected control points are not indicated for the treatment of digestion-related pathologies and are not reported to improve digestive function (Fig. 4).

The blind acupuncture procedure will be the same as in the specific acupuncture group, whereby acupuncture doctor 1 (blind) will mark the points in green marker, and acupuncture doctor 2 (blind) will disinfect the acupoint with a 70% alcohol pad and insert the needles into the marked points. A total of 10 bilateral sterile “Yu Kuang” acupuncture needles 40 mm with 30 G will be inserted perpendicularly into the depth of the muscle level with up and down stimulation and 180° rotation of the needles to both directions to generate a “De Qi” sensation. The needles will be retained for 30 minutes until removal by acupuncture doctor 2 who will not be allowed to communicate with the patients. During the session the patients will stay in a supine position. At the end of the treatment acupuncture doctors, 1 and 2 will be asked to speculate if they treat the specific or nonspecific acupuncture group to assess and maintain the integrity of the blindness in the study.

### 2.11 Drug treatment

Patients in all groups will receive metoclopramide 10 mg/8 hours in the case of poor digestion, alongside the individualized drug treatment prescribed by the ICU medical doctor as per individual patient needs.

### 2.12 Enteral feeding protocol

Nasogastric (NG) tubes will be inserted for the patients during surgery for the duration of surgery and postoperative time to service the nutritional need as per the patient prognosis/requirement. NG tubing depth and function will be fixed and checked every 24 hours and will be retained as long as the patient needs it. TEE will be calculated by the weight, height, and physical factors of the patient as assessed by the nutritionist in ICU to calculate daily nutritional needs. In postoperation day 1 ICU staff will start with a feeding amount of 30% of the TEE and gradually increase to 100% by day 4. Every 3 days the nutritionist will reassess the patient's condition (and percentage of nutrition intake).

### 2.13 Outcome measures

The main outcome measurements will be the number of days of intakes for each patient to achieve the TEE.

Secondary outcomes measures are the amount of prokinetic drugs prescribed by the ICU doctor in total dosage, the need of nasojejunal tube (5 consistent days of indigestion with conservative therapy), the need of parental nutrition (patients who cannot digest with daily NG tube drainage of >500 mL/day or severe diarrhea of >1000 mL/day), incidents of vomit or diarrhea (total number of times and volume in microliters), incidents of constipation (no stool passage in 3 days will be considered as constipation), reflux, nausea (number of times, measured by patient complains), GI bleeding (positive occult blood test of the NG tube drainage and in the stool), and incidents of fever episodes (body temperature >38°C) will also be recorded when they occur, albumin blood levels, total ICU stay, total hospital stay, total mechanical ventilation in days (a day of mechanical ventilation is at least 6 hours of mechanical ventilation in 1 day), and total mortality will also be measured as a secondary outcome.

In order to compare the effect of the control treatment, heart rate and blood pressure will be measured 1 hour before and after the acupuncture in both groups.

Acupuncturists will be asked to speculate if they treated the specific or nonspecific acupuncture group to assess and maintain the integrity of the blindness in the study (acupuncturists speculation questioner, see Supplementary Information).

### 2.14 Follow-up

**Figure 5:**
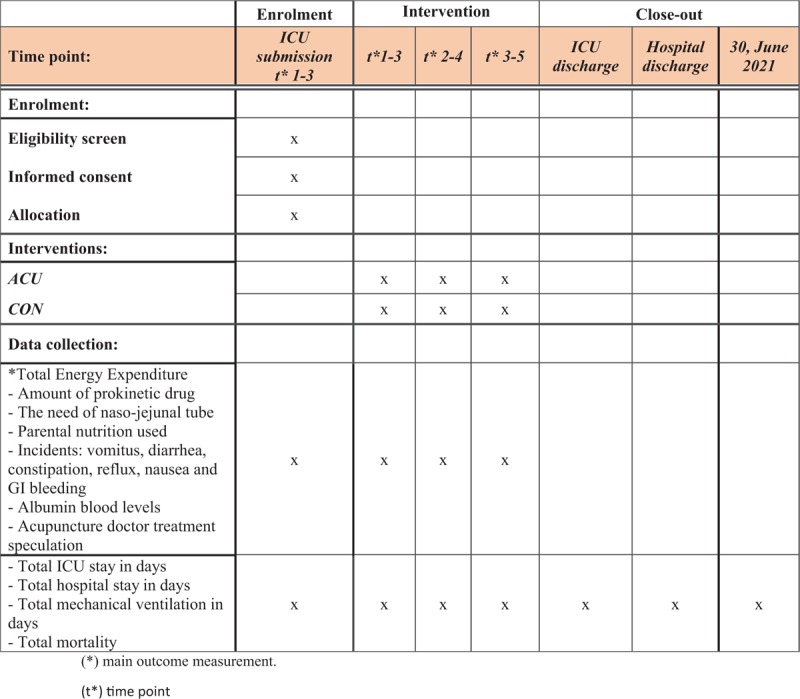
Trial schedule. schedule of enrolment, eligibility screen, informed consent, allocation, interventions, and data collection following the SPIRIT 2013 statement.^[22]^^∗^ Main outcome measurement, (*t*^∗^) time point.

Daily follow up on TEE, NG tube depth, parenteral/enteral feeding, and prokinetic drug amounts will be collected by ICU staff along with incidents of vomiting, diarrhea, constipation, reflux, nausea, and GI bleeding. After 3 days, the data collected will be used for calculating study results. Mechanical ventilation days needed and total ICU stay along with total hospital inpatient duration and mortality will be collected by the time of hospital discharge or until June 2021 (Fig. 5).

### 2.15 Adverse events

Before patients’ enrollment, the patient's legal guardian will be informed on possible acupuncture adverse events including minor bleeding, hematoma, infections, alcohol allergy, etc. Any case of acupuncture-related severe adverse events taking place, the event will be reported immediately to primary investigator and to the ethics committee, adverse events details such as treatment site, acupoint selected, patient recovery outcome, and the number of events on 1 patient will be recorded. Acupuncture will be stopped and treatment groups will be unblind. The patient will be provided with suitable ICU treatment. China medical hospital ethics committee will be in charge of deciding whether the study can continue, should be altered, or shut down.

### 2.16 Sample size calculation

The sample size was calculated using G∗power version 3.1.9.2 software, the sample size was based on data of 51 patients that visit the ICU department 9 months before the experiment and are similar to the study target population. A 95% power was calculated with an effect size of 1.68 and a calculation of 20% patient dropout rate required 14 patients in each group for a total of 28 patients.

### 2.17 Data management

In order to maintain the integrity of measured data and providing proper data reporting, patients’ data will be recorded manually as a case report form and uploaded to the hospital electronic database by ICU staff, the manual case reports will be stored in a secure location in the ICU and will be destroyed after trial completion. Regular meeting and presentations will ensure that the entire study staff is aware of the study protocol and follow it. At the beginning of the study, the principal investigator will make sure that patients enrolled are meeting inclusion criteria, the informed consent form is properly signed and all study procedures are following the study protocol. Any modification in the study protocol will be done upon agreement with China Medical University Hospital Ethics Committee and with www.clinicaltrial.gov.

### 2.18 Statistical analysis

A researcher who is blind to patient allocation will conduct the statistical analysis. Statistical data will be described as a percentage (n %) for categorical data and as mean ± standard deviations for continuous data. Continuous variables will be analyzed using the *t* test or the Mann-Whitney *U* test and categorical variables using the Chi-square (χ^2^) test or the Fisher exact test with a best applicable basis. Repeated measurements will use the generalized linear model generalized estimating equation to conduct the analysis. The analysis will be performed using the IBM SPSS Statistics version 22.0 (SPSS Inc, Chicago, IL), the results will be marked as significant at *P* value <.05.

## 3 Discussion

In critically ill patients, the incidence of indigestion can be as high as 46%; this may lead to malnutrition, increase ICU stay, and increased mortality.^[10]^ In order to treat indigestion prokinetic drugs are commonly used, although prokinetic may carry some adverse reactions.^[11]^ From data collected from our ICU department before our study, oral and hypopharyngeal cancer patients suffering from postoperative feeding intolerance will reach their TEE after 9 days with the use of prokinetic drugs, on the contrary, postoperative oral and hypopharyngeal cancer patients that do not suffer from feeding intolerance will reach their TEE in <3 days. There is a need to use a safe and effective method to improve poor digestion in critically ill cancer patients. Acupuncture has been reported to improve indigestion signs in several studies.^[19,23–25]^

For study design, we decided to conduct a double-blind design to minimize the bias. A double-blind design is unusual in acupuncture studies. Double-blind acupuncture studies are often consisting of the usage of sham needles, which carry a number of limitations; firstly, the needling depth is limited 10 mm depth which may not be ideal insertion depth in many acupoints.^[26]^ Secondly, sham needles do not allow a full up and down manipulation which may be crucial in achieving “DE QI” sensation (an important penman in successful acupuncture treatment), and thirdly, in some cases, sham needles failed to achieve a good level of acupuncturist blinding.^[27]^ Our study design involves 2 groups; when both groups are using “real” acupuncture needles at “real” acupoints. The differences between the 2 acupuncture groups are the acupoint selected. When in the treatment group (ACU) the selected acupoints are specific for the treatment of indigestion, the CON is here to present the placebo effect of acupuncture by needling acupoints that do not have any indication to treat indigestion. In both groups, the acupuncturist will perform needles stimulation that will activate the “DE QI” sensation. The acupuncturists performing the treatments will be blind to the study goal to treat indigestion, a fact that we believe will make it difficult to speculate which of the group is receiving the “real” treatment and allow us to generate blinding of the acupuncturist and patients. After a treatment session, we decided to ask the acupuncture doctors to speculate which treatment was the “real” treatment to calculate the level of doctor blindness. The patients will not be asked to speculate on the treatment nature since both groups will receive traditional medicine style acupuncture and due to the nature of ICU patients that may not be in a fully conscious state.

The acupoints selected in the treatment group (specific acupuncture group) are; ST36 (Zusanli), ST37 (Shangjuxu), ST39 (Xiajuxu), PC6 (Neiguan), and LI4 (Hegu). The acupoint, PC6 (Neiguan) main characteristics are regulating the stomach, relieve nausea and vomiting, and the point was used successfully in reducing gastric emptying.^[7,20]^ The 3 acupoints, ST36 (Zusanli), ST37 (Shangjuxu), and ST39 (Xiajuxu) are “He-Sea” points of stomach, large, and small intestines, respectively and are used to benefit those organs, the points were also a part of the acupoint combination used in a recent pilot study in improving gastric emptying.^[20]^ ST36 (Zusanli) and ST37 (Shangjuxu) also showed to improve small intestines motility in animal studies; additionally, ST36 (Zusanli) showed to increased gastric motility through a vagal pathway.^[17,28,29]^ The acupoint LI4 (Hegu) a “Yuan-Source” point of the Large Intestine channel is usually indicated for treating pain-related conditions alongside with the ability to benefit the digestive system, the points showed a beneficial effect on irritable bowel syndrome and on rats with gastric carcinectomy.^[30,31]^

The selected acupoint in the CON (nonspecific acupuncture group) are LI 15 (Jianyu), SJ 14 (JianLiao) LU3 (Tianfu), GB35 (Yangjiao), and BL 59 (Fuyang). The acupoints LI 15 (Jianyu) and SJ 14 (JianLiao) are located on the shoulder and used to treat shoulder conditions.^[32]^ LU3 (Tianfu) is used mainly for mental imbalance and for cough; GB35 (Yangjiao) is used for pain in the knee and leg; and BL 59 (Fuyang) is used for low back and thigh pain.^[33]^ None of the points in this group are used to benefit the digestive system and we believe that the points can be suitable to demonstrate the placebo effect of acupuncture treatment. An acupuncturist might speculate that the patients receiving those acupoints are suffering from shoulder pain.

## Acknowledgments

The authors would like to thank the Chinese Medicine Research Center, China Medical University for the support to our study. The authors would like to show their gratitude to Wei Tsu-Hsuan, MD and Chang Chiu-Ming, MD for performing acupuncture.

## Author contribution

**Analysis and interpretation of the data:** Ho Wen-Chao, Eyal Ben-Arie, Kao Pei-Yu

**Conceptualization:** Eyal Ben-Arie, Kao Pei-Yu.

**Data curation:** Eyal Ben-Arie, Kao Pei-Yu.

**Design:** Eyal Ben-Arie, Kao Pei-Yu, Lee Yu Chen, Ho Wen-Chao

**Formal analysis:** Ho Wen-Chao.

**Funding acquisition:** Lee Yu Chen.

**Investigation:** Eyal Ben-Arie, Kao Pei-Yu.

**Methodology:** Eyal Ben-Arie, Kao Pei-Yu, Ho Wen-Chao, Lee Yu Chen.

**Project administration:** Eyal Ben-Arie, Kao Pei-Yu, Lee Yu Chen.

**Resources:** Lee Yu Chen.

**Software:** Eyal Ben-Arie, Kao Pei-Yu, Ho Wen-Chao.

**Supervision:** Lee Yu Chen.

**Validation:** Eyal Ben-Arie, Lee Yu Chen

**Validation:** Kao Pei-Yu.

**Visualization:** Kao Pei-Yu.

**Writing – original draft:** Eyal Ben-Arie, Kao Pei-Yu.

**Writing – review and editing:** Eyal Ben-Arie, Kao Pei-Yu.
